# Iléus biliaire colique: une cause rare d’occlusion colique

**DOI:** 10.11604/pamj.2017.27.187.12238

**Published:** 2017-07-11

**Authors:** Khalid Mazine, Pierre Barsotti, Hicham Elbouhaddouti, Khalid Ait Taleb

**Affiliations:** 1 Service de Chirurgie Viscérale A (C3), CHU Hassan II Fès, Maroc; 2 Service de Chirurgie Générale Digestive et Endocrine, Hôpital Emile Müller GHR Mulhouse, France

**Keywords:** Calcul biliaire, iléus biliaire, fistule cholécystocolique, occlusion colique, Gallstone, bile ileus, cholecystocolonic fistula, colonic obstruction

## Abstract

L'iléus biliaire avec migration du calcul dans le côlon à travers une fistule cholécystocolique est rare. Le diagnostic est difficile et souvent tardif. Nous rapportons ici le cas d'une patiente de 89 ans aux antécédents d'une diverticulose du sigmoïde, qui a présenté une occlusion colique en rapport avec un iléus biliaire par migration d'un gros calcul à travers une fistule cholécysto-colique. La TDM abdominale a permis de poser le diagnostic. La chirurgie a permis d'extraire du calcul par sigmoïdotomie transformée par la suite en une sigmoïdostomie avec rétablissement ultérieur de la continuité digestive, la fistule cholécysto-duodénale n'a pas été objectivée.

## Abstract (en)

Bile ileus with migration of the gallstone into the colon through cholecystocolonic fistula is rare. The diagnosis is difficult and often late. We here report the case of a 89-year old patient with a history of sigmoid diverticular disease presenting with colonic obstruction associated with bile ileus caused by migration of a large gallstone through cholecystocolonic fistula. Abdominal CT scan allowed the diagnosis. The patient underwent surgical extraction of the gallstone with sigmoidotomy followed by sigmoidostomy with subsequent recovery of the digestive continuity. The cholecystocolonic fistula wasn’t identified.

## Introduction

L'occlusion colique par migration d'un calcul vésiculaire dans le côlon au travers d'une fistule cholécystocolique est un tableau peu fréquent d'iléus biliaire. Il doit être soupçonné chez tout malade ayant un syndrome occlusif associé à une aérobilie et une localisation éctopique d'un calcul et d'autant plus que le malade est âgé [1]. Nous rapportons ici un cas d'occlusion colique gauche compliquant une lithiase vésiculaire fistulisée dans le colon droit et qui a migré dans le colon sigmoïde. La présentation de cette observation permettra de discuter des modalités diagnostiques et thérapeutiques de cette rare entité.

## Patient et observation

**Figure 1:**
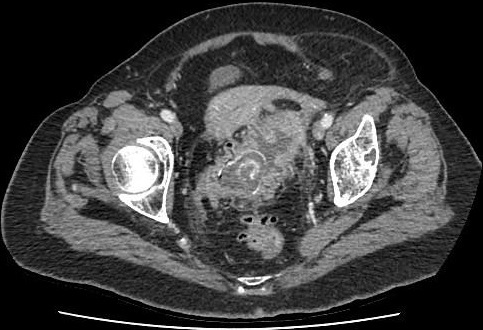
Scanner abdominal du calcul enclave

**Figure 2:**
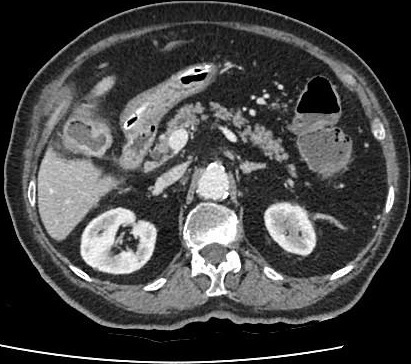
Scanner de la fistule cholecysto-colique

**Figure 3:**
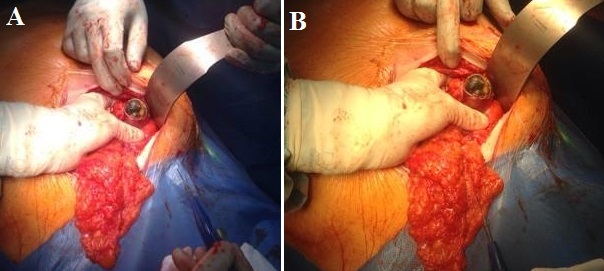
(A) Extraction du calcul par sigmoïdotomie 1; (B) Extraction du calcul par sigmoïdotomie 2

**Figure 4:**
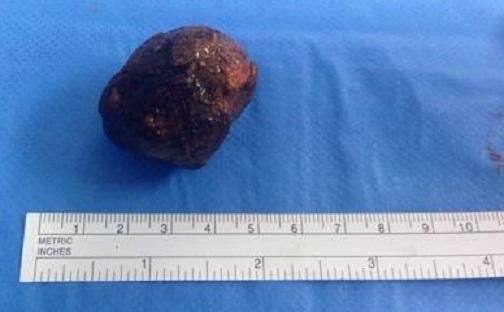
Calcul biliaire

Nous rapportons l'observation clinique d'une patiente âgée de 89 ans ayant comme antécédents pathologiques des coliques hépatiques à répétition, une diverticulose sigmoïdienne, une hypertension artérielle, une cardiopathie ischémique et un diabète sous anti diabétiques oraux. Elle a présenté un syndrome occlusif avec douleur abdominale, météorisme et arrêt des matières et des gaz. Elle a été admise aux urgences chirurgicales trois jours après le début de la symptomatologie. L'examen clinique à l'admission retrouvait une patiente consciente, stable sur le plan hémodynamique (HD) et apyrétique à 37°C. L'examen abdominal retrouvait un abdomen distendu légèrement sensible au niveau de la fosse iliaque gauche et une ampoule rectale vide au touché rectal. Un bilan biologique a été réalisé et a révélé une hémoglobine à 13g/dl, une hyperleucocytose à 15000 éléments/mm^3^ et des plaquettes à 254 000 éléments/mm^3^. L'ionogramme sanguin était normal. Après mise en condition de la patiente avec réhydratation , mise en place d'une sonde naso-gastrique et surveillance de l'état hémodynamique et de la diurèse, un scanner abdomino-pélvien a été réalisé, il a objectivé une occlusion colique à point de départ du tiers moyen du colon sigmoïde en rapport avec la présence d'une lithiase endoluminale de 4 cm de diamètre, présence d'une fistule cholécysto-colique droite de 1,7cm de diamètre (fond vésiculaire fistulisé au colon) avec présence d'air en intra vésiculaire, présence également d'une diverticulose sigmoïdienne associée (Figure 1, Figure 2). Devant le tableau d'occlusion que présente la patiente et la non amélioration elle a été prise en charge chirurgicalement, abordée par une laparotomie médiane sous ombilicale avec sigmoïdotomie extraction du calcul enclavé et transformation de la sigmoïdotomie en sigmoïdostomie latérale gauche sur baguette (Figure 3, Figure 4). La vésicule biliaire était scléro-atrophique avec une paroi complètement ratatinée collée au colon droit, la fistule cholécysto-colique a été respectée. Les suites post opératoires étaient simples avec un retour à domicile à j 7. Un rétablissement de la continuité digestive était prévu ultérieurement.

## Discussion

L'iléus biliaire est une occlusion intestinale en rapport avec la migration d'un calcul vésiculaire dans la lumière intestinale à travers une fistule, il survient chez 6 à 14% des patients ayant une fistule bilio-digéstive et représente 1 à 4% des occlusions organiques, mais peut atteindre 25% chez les personnes de plus de 65 ans, cette pathologie prédomine chez la femme âgée [1]. L'iléon terminal est la localisation la plus fréquente d'obstruction par un calcul biliaire (60 à 70%), car la valvule iléo-caecale est le lieu d'impaction le plus fréquent du calcul [2]. Le jéjunum est le site d'obstruction dans 15 à 20% des cas, et le calcul ne s'impacte dans le bulbe duodénal que dans 10% des cas. L'obstruction du colon par un calcul biliaire est exceptionnelle. L'iléus biliaire avec obstruction colique ne représente que 2 à 8% des iléus biliaires [2, 3]. Elle implique le plus souvent l'existence d'une fistule directe entre les voies biliaires et le côlon, car le calcul ne franchit qu'exceptionnellement la valvule de Bauhin. Le calcul, s'il est de grande taille, peut à lui seul, sur côlon sain, être responsable de l'occlusion. Un petit calcul peut également entraîner une occlusion sur côlon pathologique, en particulier divérticulaire par diminution du calibre colique ce qui est le cas dans notre observation [4]. Les fistules biliodigestives au travers desquelles migrent les calculs, constituent une complication peu fréquente chez les patients porteurs d'une lithiasevésiculaire biliaire (1 à 3 % avec une sex-ratio de 3 femmes pour un homme) [1]. Les localisations des fistules par ordre décroissant de fréquence sont [2]: cholécysto-duodénales: 76%; cholécysto-coliques: 15%; cholécysto-cholédociennes: 3%; multiples: 3%; cholécysto-gastriques: 2%; cholédoco-duodénales: 1%.

Il existe d'autres causes moins fréquentes de fistules entérobiliaires: l'ulcèrepeptique, les traumatismes, les causes néoplasiques et les malformations congénitales. Le mécanisme de formation des fistules est en rapport avec des épisodes répétés decholécystites lithiasiques. Il se produit une inflammation chronique, la séreuse de la vésicule biliaire devient adhérente à la séreuse du viscère en regard, pour aboutir à une érosion et à la migration du calcul [4]. Les zones les plus proches de la vésicule biliaire, comme le duodénum et l'angle colique droit, sont le plus souvent intéressées par le processus fistuleux (exceptionnellement entre la vésicule et lesigmoïde ou le côlon transverse). Les fistules cholédoco-duodénales oucholédoco-coliques sont exceptionnelles, lors d'agénésie congénitale de la vésiculebiliaire ou après cholécystectomie. L'intermittence des symptômes et l'absenced'antécédent vésiculaire connu chez la moitié des patients rendent le diagnosticdifficile [3], comme dans notre observation. À l'ASP, on peut visualiser la classique triade de Rigler (1941): occlusion avecniveaux hydro-aériques, aérobilie et calcul ectopique, mais cette triade n'est complèteque dans 25% des cas; 85% des calculs vésiculaires sont radio-transparents etl'aérobilie est inconstante (50% des cas) [5]. L'échographie est souvent peu contributive, car gênée par les gaz digestifs. Parfois, elle peut compléter, avec l'ASP, la triade de Rigler en montrant l'imagehyperéchogène avec cône d'ombre du calcul, une aérobilie et une vésicule biliairescléro-atrophique [6, 7]. La tomodensitométrie est l'examen de choix pour le diagnostic préopératoire d'un iléus biliaire [8]. C'est un examen rapide et fiable, il permet de visualiser de déceler l'aérobilie, même minime, et objective l'occlusion d'amont [5]. Dans notre observation, le scanner abdomino-pelvien était le seul examen d'imagerie pratiqué chez la patiente il a permis de poser le diagnostic en individualisant le calcul, l'occlusion colique d'amont est la fistule cholecysto-colique.

Le traitement de l'iléus biliaire colique est le plus souvent chirurgical: extraction du calcul par simple colotomie, associée ou non à la résection d'unsegment digestif nécrosé; la cholécystectomie et la cure de fistule sont discutées: non réalisées,réalisées dans un second temps opératoire ou réalisées dans le même tempsopératoire. Des cas de fermetures spontanées de fistules ont été décrits [3]; l'indication dépend en fait beaucoup de l'âge et de l'état général du sujet. Certains ont recours à la chirurgie cœlioscopique, d'autres ont rapporté des fragmentations de calculs par lithotrithie extracorporelle ou par coloscopie, notamment encas de contre-indications opératoires. L'extraction de calcul par entérotomie sans cholécystectomie semble être une méthode simple, efficace et de faible morbidité [6]. L'expulsion spontanée du calcul reste très rare [9]. La mortalité de l'iléus biliaire s'élève de 8 à 20% avec une moyenne de 14%. La mortalité opératoire est de 13%. Les complications sont fréquentes (50%): les infections postopératoires sont retrouvées dans 11 à 75% des cas, avec une moyenne à 40 %. La récidive de l'iléus biliaire survient dans 5 à 9% des cas [10, 11].

## Conclusion

L'occlusion colique secondaire à un calcul vésiculaire ayant migré au travers d'une fistule cholécystocolique reste une cause rare de syndrome abdominal aigu. Le diagnostic clinique est difficile . Le scanner contribue au diagnostic en permettant d'objectiver le calcul, l'aérobilie et parfois la fistule bilio-digestive. Le traitement chirurgical consiste en l'extraction du calcul associée ou non à une cholécystectomie et une cure de la fistule.

## Conflits d’intérêts

Les auteurs ne déclarent aucun conflit d'intérêt.
